# Type III and Not Type I Interferons Efficiently Prevent the Spread of Rotavirus in Human Intestinal Epithelial Cells

**DOI:** 10.1128/jvi.00706-22

**Published:** 2022-08-24

**Authors:** Patricio Doldan, Jin Dai, Camila Metz-Zumaran, John T. Patton, Megan L. Stanifer, Steeve Boulant

**Affiliations:** a Department of Infectious Diseases, Virology, Heidelberg University, Heidelberg, Germany; b Research Group “Cellular Polarity and Viral Infection,” German Cancer Research Center (DKFZ), Heidelberg, Germany; c Department of Biology, Indiana University, Bloomington, Indiana, USA; d Department of Infectious Diseases, Molecular Virology, Heidelberg University, Heidelberg, Germany; e Department of Molecular Genetics & Microbiology, College of Medicine, University of Florida, Gainesville, Florida, USA; Instituto de Biotecnologia/UNAM

**Keywords:** rotavirus NSP1, autocrine signaling, human intestinal epithelial cells, interferons, lambda interferon (IFN), paracrine signaling, rotavirus, type III interferon (IFN)

## Abstract

Rotavirus infects intestinal epithelial cells and is the leading cause of gastroenteritis in infants worldwide. Upon viral infection, intestinal cells produce type I and type III interferons (IFNs) to alert the tissue and promote an antiviral state. These two types of IFN bind to different receptors but induce similar pathways that stimulate the activation of interferon-stimulated genes (ISGs) to combat viral infection. In this work, we studied the spread of a fluorescent wild-type (WT) SA11 rotavirus in human colorectal cancer cells lacking specific interferon receptors and compared it to that of an NSP1 mutant rotavirus that cannot interfere with the host intrinsic innate immune response. We could show that the WT rotavirus efficiently blocks the production of type I IFNs but that type III IFNs are still produced, whereas the NSP1 mutant rotavirus allows the production of both. Interestingly, while both exogenously added type I and type III IFNs could efficiently protect cells against rotavirus infection, endogenous type III IFNs were found to be key to limit infection of human intestinal cells by rotavirus. By using a fluorescent reporter cell line to highlight the cells mounting an antiviral program, we could show that paracrine signaling driven by type III IFNs efficiently controls the spread of both WT and NSP1 mutant rotavirus. Our results strongly suggest that NSP1 efficiently blocks the type I IFN-mediated antiviral response; however, its restriction of the type III IFN-mediated one is not sufficient to prevent type III IFNs from partially inhibiting viral spread in intestinal epithelial cells. Additionally, our findings further highlight the importance of type III IFNs in controlling rotavirus infection, which could be exploited as antiviral therapeutic measures.

**IMPORTANCE** Rotavirus is one of the most common causes of gastroenteritis worldwide. In developing countries, rotavirus infections lead to more than 200,000 deaths in infants and children. The intestinal epithelial cells lining the gastrointestinal tract combat rotavirus infection by two key antiviral compounds known as type I and III interferons. However, rotavirus has developed countermeasures to block the antiviral actions of the interferons. In this work, we evaluated the arms race between rotavirus and type I and III interferons. We determined that although rotavirus could block the induction of type I interferons, it was unable to block type III interferons. The ability of infected cells to produce and release type III interferons leads to the protection of the noninfected neighboring cells and the clearance of rotavirus infection from the epithelium. This suggests that type III interferons are key antiviral agents and could be used to help control rotavirus infections in children.

## INTRODUCTION

The intestinal epithelium is composed of a monolayer of cells that acts as a primary barrier protecting the body from invading enteric pathogens. Upon infection of this barrier, enteric viruses (e.g., rotavirus, norovirus, and astrovirus) cause serious gastroenteritis in children and immunocompromised adults. Rotavirus is the main cause of gastroenteritis worldwide, accounting for approximately 40% of gastroenteritis hospitalizations in children below the age of 5 (1). More than 200,000 children die of rotavirus gastroenteritis and associated diarrhea per year (2). The first vaccines against rotavirus, Rotarix and RotaTeq, were made commercially available by GSK and Merck, respectively. These vaccines have been shown to be efficient in preventing approximately 85% of rotavirus gastroenteritis cases in low-mortality regions and about 50% in high-mortality regions (3). Similar vaccines were later developed in Asia, showing comparable efficacy. Due to these low protection rates in high-mortality regions, understanding how rotavirus replicates and spreads in human intestinal epithelial cells and how it can be controlled is critical, as it could offer novel therapeutic solutions to control rotavirus infection.

Interferons (IFNs) are the main cytokines produced by intestinal cells to fight against enteric viral infection to control virus replication and spread within the body. Upon release and binding to their specific receptors, IFNs induce a JAK/STAT-mediated signaling cascade that culminates in the production of hundreds of interferon-stimulated genes (ISGs). ISGs produce an antiviral state in both infected and noninfected neighboring cells, thereby protecting them from further virus infection (4). The human intestinal epithelium exploits two types of IFNs for its protection: type I and type III IFNs. Type I IFNs (IFN-α, IFN-β, and the less-well-characterized IFN-ɛ, IFN-τ, IFN-κ, IFN-ω, IFN-δ, and IFN-ζ) can be produced and sensed by all cell types as their heterodimeric receptor IFNAR1/IFNAR2 is ubiquitously expressed (5). Type III IFNs (IFN-λ1, -2, -3, and -4) can also be produced by all cell types, but their sensing is restricted to epithelial cells and a subset immune cells, due to the restricted expression of the IFNLR1 subunit of the IFNLR1/IL10Rβ heterodimeric receptor in these cell types (6–8). Following binding to their heterodimeric receptors, type I and III FNs induce similar signaling cascades leading to the expression of similar sets of ISGs and, thus, were originally considered to be redundant (9, 10). Nevertheless, studies have shown that type I and III IFNs display unique antiviral properties and characteristics. Type III IFNs have been suggested to be milder IFNs due to their delayed and weaker induction of ISGs. However, experiments performed in murine airway epithelium demonstrate that type III IFNs also promote tissue damage and can interfere with tissue homeostasis and repair (11). It was recently demonstrated that type III IFNs can damage the lung epithelium by promoting loss of barrier function and consequently causing susceptibility to lethal bacterial superinfections (12). Signaling downstream of the type III IFN receptor (IFNR) complex is not downregulated and allows for a sustained IFN response following type III IFN treatment (13–15). Conversely, type I IFNs lead to a faster, stronger, and shorter induction of ISGs than do type III IFNs (16–18). Interestingly, while both type I and type III IFN-mediated signaling leads to the activation of mitogen-activated protein kinases (MAPKs), only the antiviral activity of type III IFN depends on MAPKs, further highlighting the differences between these two cytokines. The model in which the type III IFN is better adapted to mucosal surfaces, providing efficient protection against viruses while limiting inflammation and tissue damage, has been recently challenged.

The differences in the ability of type I and type III IFNs to control the spread of rotaviruses in the intestinal epithelium has been extensively studied in mouse models. It was shown that mice lacking IFNLR1 exhibited a significant increase in levels of rotavirus in their villi and displayed more tissue damage than wild-type (WT) mice or mice lacking IFNAR1 (7). Furthermore, treatment of neonatal mouse intestinal epithelial cells (IECs) with type III IFNs inhibited the replication of rotavirus, whereas treatment with type I IFNs failed to do so (12). In contrast, other studies have shown that younger mice also use type I IFNs to control virus spread and replication within the intestine, while adult mice rely on type III IFNs only to control virus spread (19).

In the case of human models, these differences between type I and type III IFNs have been evaluated in both transformed cell models and primary cell models. These studies have shown that rotavirus infection induces the production of IFNs; however, each cell model induced a unique set of IFNs (IFN-λ1, IFN-λ2/3, or IFN-β1) to combat rotavirus infection (20–22). Interestingly, exogenous treatment of human intestinal epithelial cells and human intestinal organoids with either type I or type III IFNs can reduce rotavirus infection (20). These observations confirm the mostly redundant functions of these two types of IFNs, especially when provided in *trans*. However, it remains unclear which type of IFNs, when endogenously produced, protects against rotavirus infection in human intestinal epithelial cells.

Blocking type I or type III signaling in human intestinal organoids through neutralizing antibodies showed no differences in rotavirus titers after several rounds of replication, showing that although induced, IFNs were not able to restrict viral replication in these models (21). This lack of IFN ability to control rotaviruses can be due to viral mechanisms which have evolved to escape the innate immune response. Rotavirus NSP1 is known to induce the proteasomal degradation of interferon regulatory factor 3 (IRF3), IRF5, IRF7, and IRF9 and thus prevent the production of IFNs (23). Additionally, NSP1 was shown to block the activation of IRF3 in a proteasomal-degradation-independent manner by blocking its phosphorylation (24). Moreover, studies have shown that NSP1 can also prevent the activation of NF-κB and prevent STAT1/2 phosphorylation and nuclear import, thereby blocking the induction of ISGs and allowing for greater virus infection and spread (25, 26).

We have previously shown that both type I and type III IFNs were capable of controlling vesicular stomatitis virus (VSV) and reovirus infection of human intestinal epithelial cells when provided in *trans* (15). More recently, we were able to show that endogenous type III IFNs were more efficient in protecting against severe acute respiratory syndrome coronavirus 2 (SARS-CoV-2) infection than endogenous type I IFNs (27).

To determine if type III IFNs also played a key role in controlling rotavirus infection in human intestinal epithelial cells, we have taken advantage of our WT and NSP1 mutant rotaviruses expressing an UnaG fluorescent reporter (28). Using these fluorescent viruses and cells expressing a fluorescent protein under the control of the promoter region of the ISG Mx1, we were able to track virus infection and spread in WT and IFN receptor knockout (KO) cells at the single-cell level. We could correlate the efficiency of rotavirus spreading with the generation of an immune response using live cell microscopy. This study revealed that type III IFNs were produced and secreted by both WT and NSP1 mutant rotavirus-infected cells and that type III IFNs were able to control the spread of both rotaviruses in human intestinal epithelial cells. Type I IFNs were induced only following infection by the NSP1 mutant rotavirus but did not produce enough protection in bystander cells to control rotavirus spread. These results highlight the key functions that endogenous type III IFNs play in controlling virus spread within the human intestinal epithelium and suggest that they could act as key antivirals to combat rotavirus infection.

## RESULTS

### NSP1 is key to control interferon induction in human intestinal epithelial cells.

**FIG 1:**
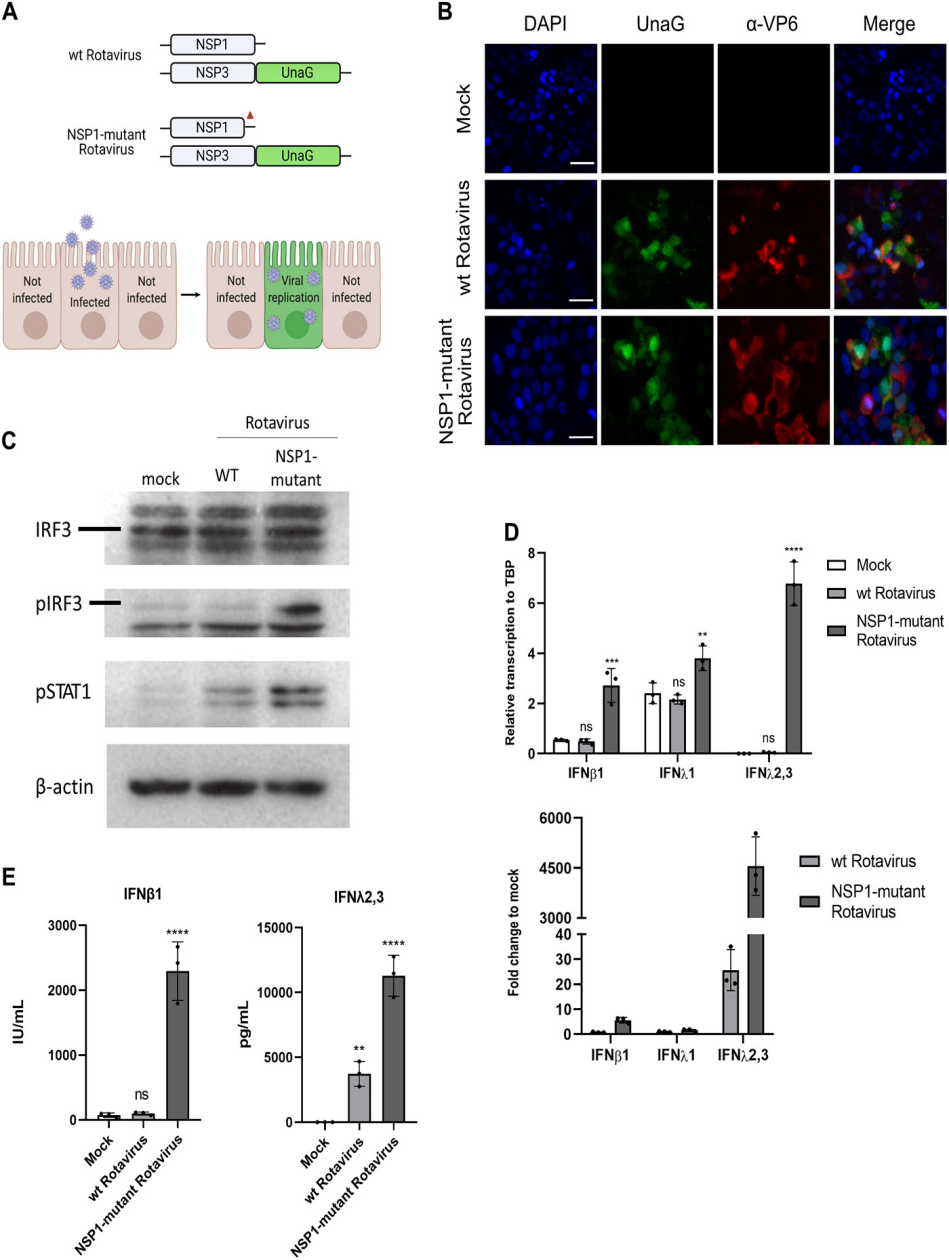
UnaG-tagged rotavirus induces an IFN-mediated immune response in T84 cells. (A) Schematic of fluorescent WT and NSP1 mutant rotaviruses which contain UnaG fused to NSP3 and thus fluoresce upon translation of viral proteins. (B) T84 cells were infected with either UnaG-expressing WT or NSP1 mutant rotavirus at an MOI of 1 (as determined in MA104 cells) and fixed at 24 hpi. Indirect immunofluorescence was carried out targeting the viral capsid protein VP6 (magenta). Nuclei were stained with DAPI (blue). Representative images are shown. Scale bar, 50 μm. (C) T84 cells were infected with WT and NSP1 mutant rotavirus at an MOI of 5. At 24 hpi, cells were harvested and proteins were analyzed by Western blotting. A representative image is shown. (D) T84 cells were infected with WT and NSP1 mutant rotaviruses. At 24 hpi, RNA was extracted and the upregulation of IFN-β1, IFN-λ1, and IFN-λ2/3 was analyzed by qRT-PCR. Error bars indicate SDs (*n* = 3 biological replicates). n.s., not significant. *, *P* < 0.05; **, *P* < 0.01; ***, *P* < 0.001; ****, *P* < 0.0001 (ordinary one-way ANOVA with Dunnett’s multiple-comparison test using noninfected cells as a reference). (E) T84 cells were infected at an MOI of 1 with either WT or NSP1 mutant rotavirus, and the levels of IFNs (IFN-β1 or IFN-λ1 to -3) produced were measured by adding the supernatants to HEK-Blue reporter cells at 24 hpi. Error bars indicate SDs (*n* = 3 biological replicates). *, *P* < 0.05; **, *P* < 0.01; ***, *P* < 0.001; ****, *P* < 0.0001 (ordinary one-way ANOVA with Dunnett’s multiple-comparison test using noninfected cells as a reference).

To determine how human intestinal epithelial cells respond to rotaviruses, WT and NSP1 mutant SA11 rotaviruses encoding the green fluorescent protein UnaG were used. These viruses carry UnaG fused to their NSP3 gene, and thus, the fluorescent protein is visualized only after viral replication and translation of viral proteins (Fig. 1A) (28). The NSP1 mutant rotavirus contains a deletion of 17 amino acids at the C terminus of NSP1, hindering its ability to block the induction of interferons (29). To validate that the fluorescent protein UnaG can be used as a robust reporter of rotavirus infection in human intestinal epithelial cells, T84 colorectal carcinoma cells were infected with WT or NSP1 mutant rotavirus at a multiplicity of infection (MOI) of 1. At 24 h postinfection (hpi), cells were fixed, infected cells were detected by immunofluorescence staining against the viral capsid protein VP6, and the VP6 immunostaining was correlated with the UnaG signal. The results showed that all cells that were positive for the viral capsid protein VP6 were also positive for the UnaG fluorescent protein, confirming that UnaG can act as a robust reporter for rotavirus infection (Fig. 1B). To confirm that NSP1 could block the activation of IRF3 and the subsequent mounting of an innate immune response, we infected T84 cells with the WT and NSP1 mutant rotavirus at an MOI of 5. At 24 hpi, cells were harvested and analyzed by Western blotting using antibodies directed against IRF3, phospho-IRF3, and phospho-STAT1. Beta-actin was used as a loading control. The results showed that the levels of IRF3 remained unchanged in WT and NSP1 mutant rotavirus-infected cells compared to those in mock-infected cells (Fig. 1C). Importantly, NSP1 mutant rotavirus-infected cells showed a higher phosphorylation of IRF3 and STAT1 than did WT rotavirus-infected cells (Fig. 1C). These findings are consistent with previous observations that rotavirus NPS1 can block activation of IRF3 in a proteasome-independent manner (24). To evaluate the extent by which WT and NSP1 mutant rotaviruses induce the production of IFNs, T84 cells were infected with either virus at an MOI of 1 and at 24 hpi, the upregulation of type I (IFN-β1) or type III IFNs (IFN-λ1 to -3) was evaluated by reverse transcription-quantitative PCR (qRT-PCR). The results showed that WT rotaviruses did not induce IFN-β1 nor IFN-λ1, as shown by the relative expression of IFNs compared to the housekeeping TATA box-binding protein (TBP) (Fig. 1D, top) or the fold change of IFN transcript level compared to that in mock-infected cells (Fig. 1D, bottom). Interestingly, transcription of IFN-λ2/3 was found to be upregulated in cells infected with WT rotavirus (Fig. 1D). Infection with the NSP1 mutant rotavirus resulted in a moderate induction of IFN-β1 and IFN-λ1 and a strong upregulation of IFN-λ2/3 transcription (Fig. 1D). These findings confirm that NSP1 can efficiently interfere with IFN production in rotavirus-infected cells.

To detect the presence of IFNs in the supernatant of rotavirus-infected cells, cells were infected at an MOI of 1 with either WT or NSP1 mutant rotavirus and the supernatants were collected 24 hpi. Supernatants were then added to HEK-Blue reporter cells genetically modified to express an alkaline phosphatase under the control of the ISG54 promoter region. At 24 h posttreatment, a colorimetric assay was carried out to measure the amount of IFNs released by rotavirus-infected cells. In accordance with our qRT-PCR results, no IFN-β1 was produced by cells infected with WT rotavirus, whereas it was readily detectable in the supernatant of cells infected with the NSP1 mutant rotavirus (Fig. 1E, left). In contrast, IFN-λ1 to -3 were detectable in the supernatants of both WT and NSP1 mutant rotavirus-infected cells, though to a much larger extent upon NSP1 mutant rotavirus infection (Fig. 1E, right). Together, these results show that NSP1 is key to control interferon production but that while it can completely block the production of type I IFNs, it appears to only partially impact the production of type III IFNs.

### Exogenous treatment with interferons blocks rotavirus infection of human intestinal epithelial cells.

**FIG 2:**
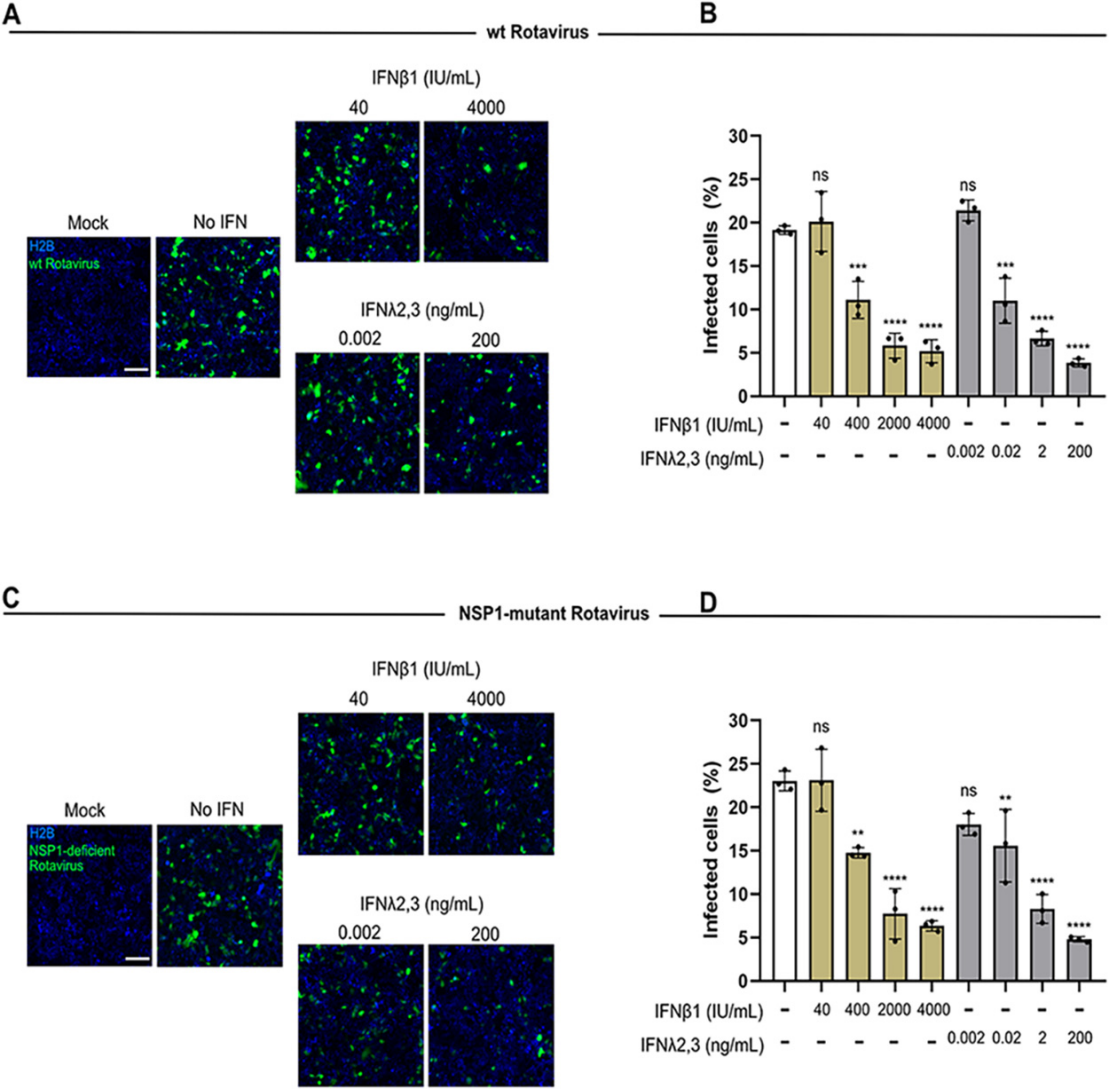
Both type I and type III IFNs can prevent rotavirus infection when added exogenously. H2B-mCherry T84 cells were pretreated with the indicated concentrations of IFN-β1 and IFN-λ2/3 for 16 h and were subsequently infected with rotavirus containing UnaG fused to NSP3. At 16 hpi, infection was analyzed by fluorescence microscopy. (A) Representative images of WT rotavirus infection by fluorescence microscopy. Scale bar, 100 μm. (B) Quantification of the percentage of infected cells. (C) Same as panel A except for NSP1 mutant virus. Scale bar, 100 μm. (D) Same as panel B except for NSP1 mutant virus. Error bars indicate SDs (*n* = 3 biological replicates). Symbols are as for Fig. 1.

To confirm whether exogenous addition of type I (IFN-β1) or type III (IFN-λ2/3) IFNs can prevent rotavirus infection, human intestinal epithelial cells were treated with increasing concentrations of either IFN for 16 h prior to infection. Following IFN treatment, WT and NSP1 mutant rotaviruses were added to T84 cells and virus infection was assayed 16 hpi by fluorescence microscopy (Fig. 2A and C). The results showed that both type I and III IFNs were able to control WT rotavirus infection in a dose-dependent manner (Fig. 2A and B). Interestingly, NSP1 mutant rotavirus displayed a sensitivity to type I and III IFNs similar to that of WT virus (Fig. 2C and D). These results suggest that T84 cells can use both type I and type III IFNs to control rotavirus infection when provided in *trans*. As WT and NSP1 mutant viruses are similarly sensitive to interferons, these data show that NSP1 mostly interferes with production of IFN (Fig. 1) but does not control antiviral signaling downstream of the IFN receptor.

### IFN-λ is essential to control the spread of rotavirus in human intestinal epithelial cells.

**FIG 3:**
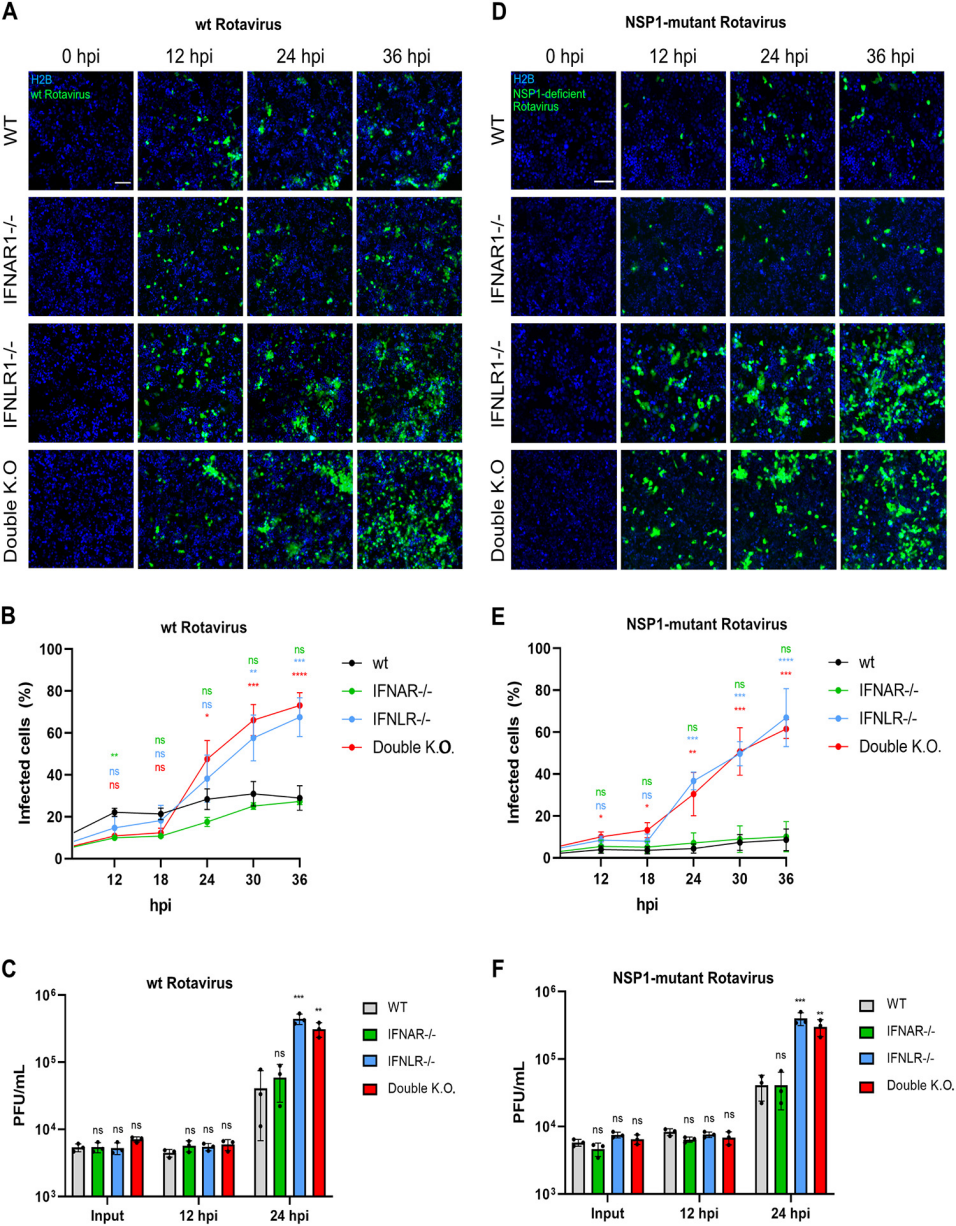
Rotavirus efficiently spreads in cells lacking a functional IFNLR1. WT, IFNAR1^−/−^, IFNLR1^−/−^, and double KO T84 cells transduced with an H2B-mCherry nuclear tag (blue) were infected with rotavirus at an MOI of 0.1 (as determined in MA104 cells). Infection was followed by live fluorescence microscopy for 36 hpi, and images were acquired every 1 h. (A) Fluorescent images of WT rotavirus infection and spread at indicated time points. Scale bar, 100 μm. (B) Quantification of the percentage of infected cells in panel A over time. (C) Supernatants from cells infected at an MOI of 2 were collected and infectious *de novo* virus was evaluated by plaque assay. (D) Same as panel A except for the NSP1 mutant virus. Scale bar, 100 μm. (E) Same as panel B except for the NSP1 mutant virus. (F) Same as panel C except for the NSP1 mutant virus. Error bars indicate SDs (*n* = 3 biological replicates). Symbols are as for Fig. 1 but with significance differences determined by multiple *t* tests using WT T84 cells as a reference.

While both type I and III IFNs can block rotavirus infection in human intestinal epithelial cells (Fig. 2), our data show that NSP1 is only able to completely block type I IFN production (Fig. 1). This suggests that in human intestinal epithelial cells, type III IFN plays a central role in controlling rotavirus infection. To determine if the endogenously produced type I and type III IFNs are capable of controlling rotavirus infection, we employed T84 cells which had been depleted of either the type I IFN receptor (IFNAR^−/−^), the type III IFN receptor (IFNLR^−/−^), or both receptors (dKO). To track rotavirus infection and spread, WT and IFN receptor mutant T84 cells were transduced with the fluorescent nuclear tag H2B-mCherry and cells were infected at an MOI of 0.1 with either the WT or the NSP1 mutant rotavirus encoding the fluorescent reporter UnaG. Cells were monitored at 1-h intervals for 36 h by live-cell fluorescence microscopy. The results showed that loss of the type I IFN receptor (IFNAR^−/−^) did not impact WT rotavirus infection and spread in human intestinal epithelial cells (Fig. 3A to C). Importantly, cells lacking the type III IFN receptor (IFNLR^−/−^) or both receptors (dKO) supported greater infection and spread of WT rotavirus, indicating that endogenous type III IFNs are key antiviral cytokines to combat rotavirus infection (Fig. 3A and B). The findings were similar when measuring production of infectious *de novo* virus at 24 hpi using plaque assay (Fig. 3C). These results are in line with our previous observation that WT rotavirus induces the production of only IFN-λ2/3 and not IFN-β1 (Fig. 1D and E). Similar results were obtained when tracking the infection spread of the NSP1 mutant rotavirus. NSP1 mutant rotavirus could more efficiently spread in cells lacking the type III IFN receptor or cells lacking both IFN receptors (Fig. 3D and E). Similarly, we found that removal of the IFNLR or of the IFNAR and IFNLR resulted in a significantly higher production of *de novo* infectious rotavirus at 24 hpi as measured by plaque assay (Fig. 3F). Importantly, due to the higher levels of IFNs being produced following infection with the NSP1 mutant rotavirus (Fig. 1), lower levels of infected WT and IFNAR1^−/−^ cells were observed throughout the whole time course than in infections with WT rotavirus (Fig. 3B versus Fig. 3E).

Interestingly, although NSP1 mutant rotavirus infection led to the production of IFN-β1 (around 2,000 IU/mL [Fig. 1D]), high levels of infection were still observed in the IFNLR1^−/−^ cell line (Fig. 3E). As similar amounts of IFN-β1 provided in *trans* led to a reduction of rotavirus infection (Fig. 2C and D), our data suggest that endogenous IFN-β1 does not play a key role in controlling rotavirus infection. Altogether, these results show that endogenously produced type III IFNs following rotavirus infection are key to control rotavirus spread in human intestinal epithelial cells.

### Human intestinal epithelial cells use type III IFNs to induce an antiviral state.

**FIG 4:**
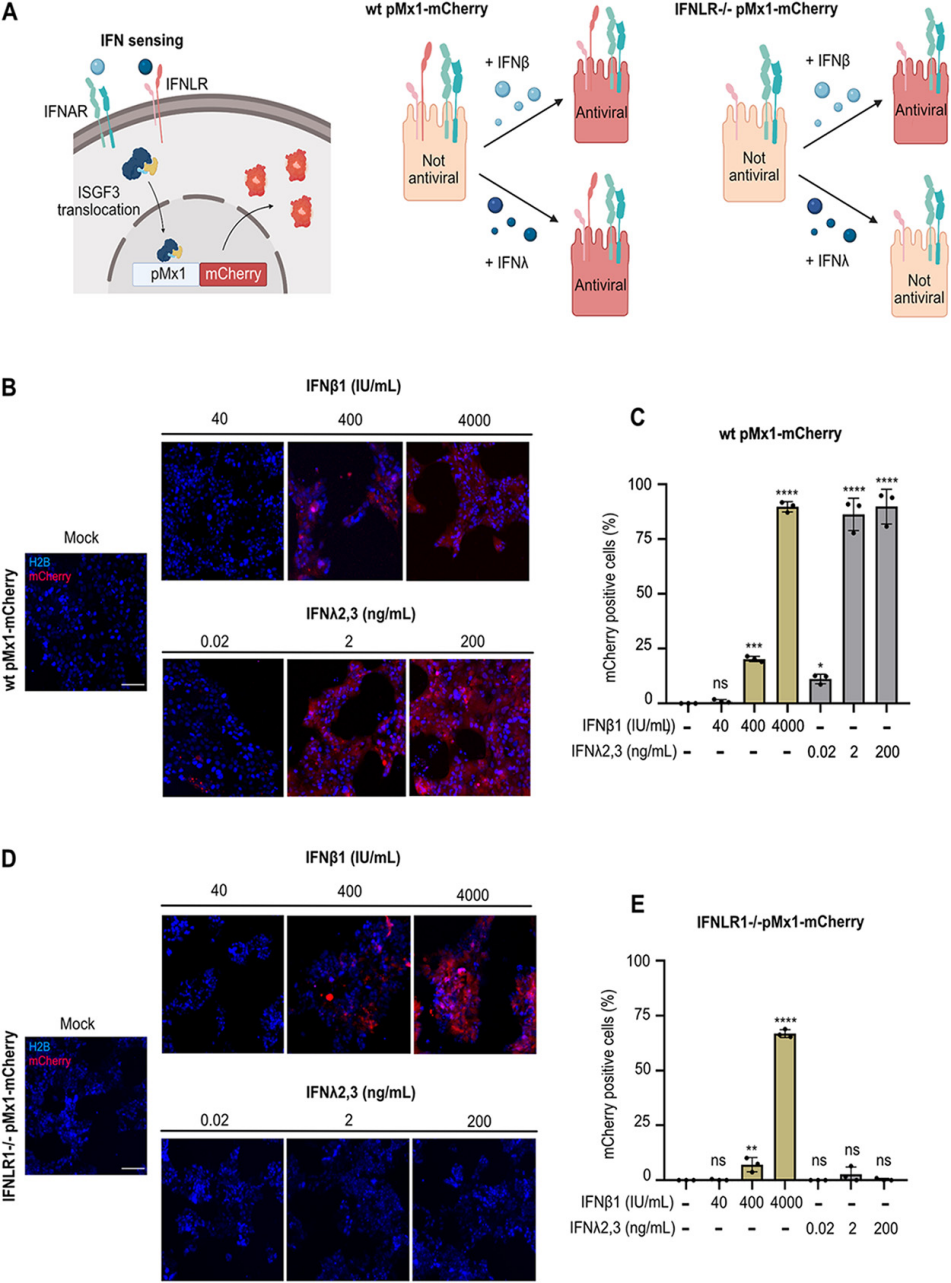
The pMx1-mCherry T84 cell lines serve as reporters of IFN sensing. (A) Schematic showing a T84 cell carrying the pMx1-mCherry IFN-sensing reporter cell line (left). WT T84 pMx1-mCherry cells express mCherry upon sensing of either type I or type III IFNs (middle), and IFNLR^−/−^ pMx1-mCherry cells express mCherry only upon sensing of type I IFNs (right). (B) WT pMx1-mCherry T84 cells were treated with increasing concentrations of either IFN-β1 or IFN-λ2/3 for 24 h. Representative fluorescent images are shown. Scale bar, 100 μm. (C) Quantification of the percentage of Mx1-mCherry cells from panel B. (D) IFNLR^−/−^ pMx1-mCherry T84 cells were treated with increasing concentrations of either IFN-β1 or IFN-λ2/3 for 24 h. Representative fluorescent images are shown. Scale bar, 100 μm. (E) Quantification of the percentage of Mx1-mCherry cells from panel D. Error bars indicate SDs (*n* = 3 biological replicates). Symbols are as for Fig. 1.

**FIG 5:**
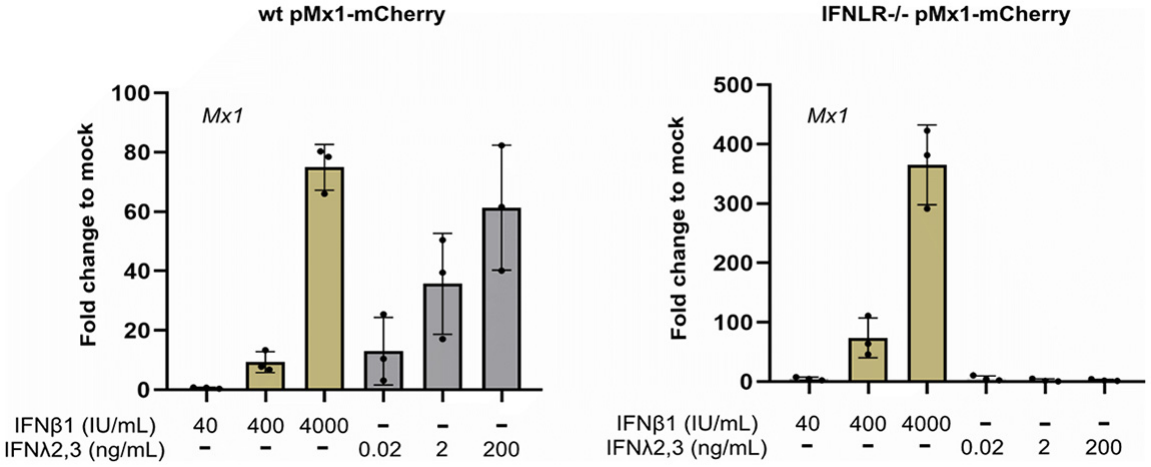
Endogenous levels of Mx1 match the activation of the pMx1-mCherry reporter. WT pMx1-mCherry and IFNLR^−/−^ pMx1-mCherry T84 cells were treated with different concentrations of IFN-β1 and IFN-λ2/3. At 24 h after treatment, RNA was harvested and qRT-PCR was performed to measure Mx1 transcript levels.

To visualize the magnitude and timing of the antiviral response elicited by type I and type III IFNs, we took advantage of an Mx1-mCherry reporter. This reporter was built by cloning the red fluorescent protein mCherry after the promoter region of the ISG Mx1, and thus its fluorescence is visualized only upon IFN-mediated signaling (Fig. 4A, left). WT and IFNLR1^−/−^ cell lines stably carrying the Mx1-mCherry reporter were generated by lentiviral transduction and single-cell cloning. WT pMx1-mCherry cells are used to determine the antiviral state induced by the presence of either type I or type III IFNs (Fig. 4A, middle), while the IFNLR1^−/−^ pMx1-mCherry cell line can report only an antiviral state induced by type I IFNs (Fig. 4A, right). WT and IFNLR1^−/−^ Mx1-mCherry cell lines were transduced with the fluorescent nuclear tag H2B-mTurquoise2 to visualize all cells (infected, noninfected, IFN-responsive, and non-IFN-responsive cells) during live experiments. To test the efficacy of our various reporter cell lines at sensing and responding to IFNs, WT and IFNLR1^−/−^ Mx1-mCherry cell lines were treated with three different concentrations of either IFN-β1 or IFN-λ2/3. At 24 h poststimulation, the number of Mx1-mCherry-positive cells was evaluated using fluorescence microscopy (Fig. 4B and D). Quantification of Mx1-mCherry-positive cells confirmed that the WT pMx1-mCherry cell line was activated by both type I and III IFNs (Fig. 4B and C). Furthermore, increasing concentrations of either type I or type III IFN led to an increased number of Mx1-mCherry-positive cells (Fig. 4B and C). Quantification showed that high concentrations of IFN-β1 induced the activation of the Mx1-mCherry reporter in approximately 90% of the cells (Fig. 4C). Concentrations of IFN-λ2/3 above 2 ng/mL were enough to activate the reporter in most cells (approximately 90%). Importantly, only IFN-β1 activated the pMx1-mCherry reporter in IFNLR^−/−^ cells, further confirming its specificity for type I IFNs (Fig. 4D and E). Quantification showed that the IFNLR^−/−^ pMx1-mCherry cell line induced a slightly smaller number of mCherry-positive cells upon treatment with IFN-β1 than for the WT pMx1-mCherry cell line (Fig. 4E). To confirm that the fluorescent signal generated by the reporter was in line with ISG transcript levels, WT and IFNLR^−/−^ pMx1-mCherry cells were treated with IFN-β1 or -λ2/3. At 24 h poststimulation, RNA was extracted, and qRT-PCR revealed a similar upregulation of the endogenous Mx1 transcript, validating the accuracy of the fluorescently tagged promoter (Fig. 5).

**FIG 6:**
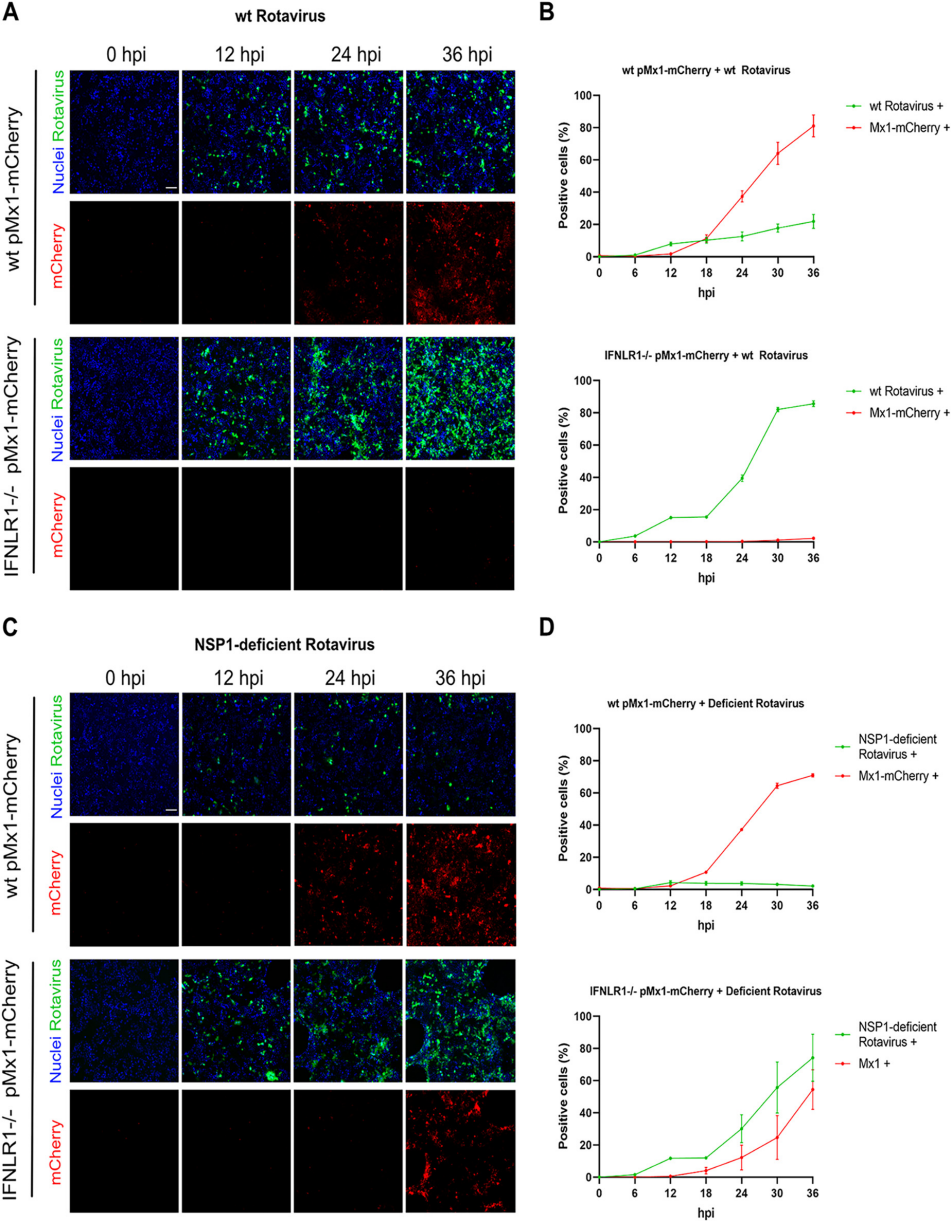
IFN-λ efficiently induces an antiviral response in T84 cells. WT pMx1-mCherry cells and IFNLR^−/−^ pMx1-mCherry cells were infected with either WT rotavirus (A and B) or NSP1 mutant rotavirus (C and D) at an MOI of 0.1 (calculated in MA104 cells). (A) Representative fluorescent images of WT rotavirus infection of WT pMx1-mCherry cells and IFNLR^−/−^ pMx1-mCherry at the indicated time points. All cells carry the H2B-mTurquoise2 nuclear tag (blue) for quantification. Scale bar, 100 μm. (B) Quantification of the number of rotavirus-infected cells and mCherry-positive cells from panel A. (C) Representative fluorescent images of NSP1 mutant rotavirus infection of WT pMx1-mCherry cells and IFNLR^−/−^ pMx1-mCherry at the indicated time points. All cells carry the H2B-mTurquoise2 nuclear tag (blue) for quantification. Scale bar, 100 μm. (D) Quantification of the number of rotavirus-infected cells and mCherry-positive cells from panel C. Error bars indicate SDs (*n* = 3 biological replicates).

To investigate whether infection with WT or NSP1 mutant rotavirus induces a type I or type III IFN-mediated antiviral response, WT pMx1-mCherry and IFNLR1^−/−^ pMx1-mCherry cells were infected at an MOI of 0.1 and UnaG (rotavirus)- and mCherry (ISG)-positive cells were quantified over time. The results showed that WT rotavirus induced the production of Mx1-mCherry in WT cells only (Fig. 6A). No activation was observed following WT rotavirus infection of IFNLR1^−/−^ pMx1-mCherry cells (Fig. 6A). These results are in line with our previous observations that only IFN-λ2/3 was produced following WT rotavirus infection and suggest that the Mx1-mCherry cells found in the WT pMx-1-mCherry cells were due to type III IFN alone (Fig. 1 and 6B). Quantifications showed that 40% of WT pMx1-mCherry cells were mounting an antiviral response at 24 hpi, which is when a second round of infection would take place. This percentage of IFN-responding cells could explain the limited ability of the virus to continue spreading in WT cells (Fig. 6B, top). After 36 hpi, approximately 80% of the cells were in an antiviral state, highlighting the capacity of IFN-λ2/3 in establishing an antiviral response in intestinal epithelial cells. Conversely, there were almost no mCherry-positive IFNLR1^−/−^ pMx1-mCherry cells throughout the time course, confirming that type I IFNs were not key to mediate the antiviral response observed upon rotavirus infection. This lack of an antiviral response led to a sharp increase in WT rotavirus infection and spread in IFNLR1^−/−^ pMx1-mCherry cells (Fig. 6B, bottom).

Similar to the case with WT rotavirus, NSP1 mutant rotavirus infection of WT pMx1-mCherry cell line showed an induction of Mx1-mCherry signal and a subsequent low level of virus spread. The NSP1 mutant virus led to an induction of 40% mCherry-positive cells at 24 hpi, which further increased to 85% at 36 hpi (Fig. 6C and D, top). Interestingly, unlike the WT rotavirus, the NSP1 mutant rotavirus led to an induction of Mx1-mCherry in the IFNLR1^−/−^ pMx1-mCherry cells at late time points, indicating that type I IFNs were secreted following infection (Fig. 6C and D, bottom). This induction of type I IFNs is in line with our previous qPCR and secreted-IFN data (Fig. 1D and E). The percentage of Mx1-mCherry-positive cells was found to be reduced in IFNLR^−/−^ cells compared to WT cells, and this led to a larger amount of rotavirus-infected cells. These results suggest that type III IFNs can induce an antiviral state in noninfected cells before a second round of infection can take place. In contrast, when produced in the context of the NSP1 mutant rotavirus, type I IFNs do not seem to induce a sufficient antiviral response to control rotavirus spread. Altogether, our results show that upon rotavirus infection, human intestinal epithelial cells produce type III IFNs and rely on this cytokine to control rotavirus spread within the epithelium.

## DISCUSSION

The differences between type I and type III IFNs in their ability to control rotavirus infection and replication have been mainly studied in murine models and in two-dimensional (2D) and 3D *in vitro* models (7, 20, 21). These studies have shown that although type III IFNs are strongly upregulated upon rotavirus infection, pretreating cells with any type of IFN can prevent infection. These approaches have limitations, as they do not necessarily reveal the importance of endogenous type I and type III IFNs in controlling the spread of rotavirus. Importantly, it remains unclear which IFNs endogenously protect human cells against rotavirus infection. In this study, we have combined live-cell fluorescence microscopy with IFN receptor knockout cell lines to thoroughly track the spread of a fluorescent rotavirus and the activation of an antiviral state in the presence or absence of either type I or type III IFNs.

Murine intestinal cells show upregulation of both type I and type III IFNs upon rotavirus infection, but the sensing of IFNs is limited to different cell types, with the lamina propria responding to type I IFNs and epithelial cells to type III IFNs (7, 12). In the case of human models, studies trying to elucidate the importance of type I and type III IFNs during rotavirus infection and spread have focused on the upregulation of IFN transcripts. The colorectal carcinoma cell line Caco-2, for example, exhibits a strong upregulation of IFN-λ1 upon infection, as well as small amounts of type I IFNs (20). Similarly, human intestinal organoids show induction of mainly IFN-λ1 and IFN-λ2 upon rotavirus infection and lower levels of type I IFNs (21). In contrast, the human colon cancer cell line HT29 shows a significant upregulation of IFN-β transcripts upon infection. We have observed that in the case of the T84 colorectal cancer cell line, WT SA11 rotavirus induces mainly the production of IFN-λ2/3, and an upregulation of type I IFNs could not be detected (Fig. 1E). The rotavirus protein NSP1 has been shown to efficiently block the production of both type I and type III IFNs (30–32), and thus, we hypothesized that NSP1 plays a role in controlling the activation of the type I IFN production. When comparing the innate immune response elicited in T84 cells by an NSP1 mutant rotavirus, we observed a stronger upregulation of IFN-λ2/3 than the one induced by WT virus, and significant amounts of IFN-λ1 and IFN-β1 were also detected (Fig. 1). These results highlight the possibility that type III IFNs may be regulated through a slightly different pathway that could have evolved to partially escape rotavirus viruses’ halt of type I IFN production. Moreover, it is also possible that small amounts of type III IFNs are produced before the NSP1-mediated shutdown of the innate system takes place and that this amount is enough to establish an antiviral response in the culture. Although IECs seem to favor type III IFNs, type I IFNs are of great importance to prevent dissemination of enteric viruses to other tissues. Mice lacking functional type I IFN receptors have shown increased reovirus infection in their lamina propria, and this was also the case for norovirus (33, 34). It is not surprising, then, that rotavirus has evolved tools to efficiently block type I IFNs, while IECs have evolved to favor type III IFNs and combat viral replication in the epithelium. Importantly, previous studies could not detect the presence of IFNs in the supernatant of infected cells, although an upregulation of transcripts was observed (20, 21). We took advantage of the HEK-Blue reporter cell lines and knocked out the IFNLR1 on them to generate a reliable assay to measure specifically the presence of either type I or type III IFNs (see Materials and Methods). These cell lines were used to successfully detect the presence of both types of IFNs in infected cells, which has not been possible in the past. This represents an important tool for elucidating the differences between type I and type III IFN-mediated antiviral responses, since viral shutdown can occur at the translation step or during IFN sensing, although upregulation of both transcripts is detected.

Previous studies have shown that type I and type III IFNs can elicit similar antiviral states when added in *trans* and thus protect cells from rotavirus infection to comparable levels (20, 21). In accordance with these studies, we could observe that pretreatment of T84 cells with either type I or type III IFNs is efficient in preventing infection (Fig. 2), showing that cells are able to respond to these types of IFNs at least when added in *trans*. Nevertheless, the timings and concentrations of IFNs required to prevent viral spread may differ in the context of an infection and therefore cannot be evaluated with this setup. To better characterize the importance of type I and type III IFNs in preventing viral spread along the intestinal epithelium, we took advantage of previously generated T84 cells lacking functional IFN receptors (15). These cell lines allow for direct comparison with experiments carried out in IFNR KO mice and elucidate at what stage and to what extent one type of IFN is important to hinder viral replication/infection. Tracking the spread of WT and NSP1 mutant rotavirus in WT, IFNAR1^−/−^, IFNLR1^−/−^ and double KO cells through live-cell imaging showed that only cells able to respond to type III IFNs could control viral spread (Fig. 3). As observed through qRT-PCR, WT rotavirus efficiently blocked the production of type I IFNs, and thus, it is likely that the small amounts of IFN-λ2/3 produced were enough to prevent a second round of infection. Moreover, infection with the NSP1 mutant rotavirus showed almost no new infections after the first round, likely due to the induction of larger amounts of IFNs (Fig. 3). Previous work has shown that type I IFNs elicit a fast and acute immune response, whereas type III IFNs elicit a response in a delayed and sustained manner (35). To evaluate whether this was the case in our setup, we took advantage of WT and IFNLR1^−/−^ T84 cells transduced with the pMx1-mCherry fluorescent reporter. Interestingly, a similar activation of the pMx1-mCherry fluorescent reporter during infection with either the WT or the NSP1 mutant rotavirus in WT cells was observed (Fig. 6), highlighting that indeed small amounts of IFN-λ2/3 may be enough to prevent viral spread. Surprisingly, the NSP1 mutant rotavirus was able to activate the pMx1-mCherry reporter in IFNLR1^−/−^ cells at late time points, showing a delayed activation of ISGs by type I IFNs that could not prevent viral spread (Fig. 6C and D, bottom). These results show kinetics opposite those observed with IFNs added in *trans* (35), with type III IFNs acting faster than type I IFNs to establish an antiviral state during rotavirus infection. It is possible, then, that large amounts of type I IFNs need to accumulate to elicit an antiviral response in IECs, since pretreatment of T84 cells with IFN-β before infection showed that these cells are indeed able to sense type I IFNs efficiently and control viral replication (Fig. 2).

The ability of type I and type III interferons to prevent the replication and spread of enteric viruses along the intestinal epithelium has been investigated in the past. These studies have focused on the use of murine models lacking specific interferon receptors or analysis of the immune response mounted by bulk populations of commercial cell lines upon infection. In this study, we utilized fluorescent reporters and live-cell fluorescence microscopy to obtain a visual understanding of the timings and extent that these types of interferon elicit in intestinal epithelial cells during rotavirus infection and spread. We could observe that type III IFNs established an antiviral state in a large number of cells very efficiently, even in the presence of IFN antagonist viral protein NSP1. On the hand, type I IFNs could be produced only in the absence of NSP1, and the antiviral state established by these cytokines was delayed and not sufficient to prevent rotavirus spread. Taking advantage of fluorescent reporters, it was possible to track how even when a low number of cells is infected, most of the cells in the culture can rapidly go into an antiviral state, highlighting the importance of the paracrine function of type III IFNs in preventing rotavirus spread. We hypothesize that intestinal epithelium favors type III IFNs over type I IFNs, since their production is not completely shut down by rotavirus and small quantities seem to be sufficient to upregulate ISGs and promote an antiviral state. In conclusion, our work highlights the central function of endogenous type III IFN in protecting intestinal epithelial cells against rotavirus infection. This activity could be exploited as antiviral prophylactic approaches to treat patients with severe/chronic rotavirus infection.

## MATERIALS AND METHODS

### Cells and viruses.

WT T84 human colon carcinoma cells (ATCC CCL-248) and derivative IFNAR1^−/−^, IFNLR1^−/−^, and IFNAR/IFNLR1^−/−^ cells (30) were maintained in a 50:50 mixture of Dulbecco’s modified Eagle’s medium (DMEM) and F-12 (Gibco) supplemented with 10% fetal bovine serum (FBS) and 1% penicillin-streptomycin (Gibco). T84 cells expressing H2B-mTurquoise2, H2B-mCherry, and pMx1-mCherry were generated via lentiviral transduction. The pMx1-mCherry construct was a kind gift from Ronald Dijkman (University of Bern).

MA104 cells were maintained in minimum essential medium (MEM; Gibco) supplemented with 10% fetal bovine serum, 1% penicillin-streptomycin (Gibco), and 2 mM l-glutamine (Gibco).

WT and NSP1 mutant rotaviruses were amplified, semipurified, and titrated through a plaque assay in MA104 cells as previously reported (36).

IFN-α/β reporter HEK 293 cells (InvivoGen; hkb-ifnab) and IFN-λ reporter HEK 293 cells (InvivoGen; hkb-ifnl) were maintained in Iscove’s modified Dulbecco’s medium (IMDM; Gibco) supplemented with 10% fetal bovine serum and 1% penicillin-streptomycin (Gibco).

### Viral infections.

All rotavirus infections were performed at the MOIs indicated. Rotavirus was activated at 37°C for 30 min in serum-free medium containing 2 μg/mL of trypsin from bovine pancreas (Sigma; T1426). Cells were washed twice with serum-free medium, virus was added to cells, and cells were incubated for 1 h at 37°C. Following the incubation, virus was removed, cells were washed twice with serum-free medium, and phenol red-free medium containing 2 μg/mL of porcine trypsin was added back to the cells for the duration of the experiment. Production of new viral particles was quantified through plaque assays as previously described (36).

### Antibodies and reagents.

For immunofluorescence experiments, mouse monoclonal antibody against rotavirus capsid protein VP6 (Santa Cruz Biotechnology; sc-101363) was used at 1:500 for immunofluorescence. Secondary antibody conjugated with Alexa Fluor 647 (AF647; Molecular Probes) directed against the animal source was used at 1:1,000. For Western blot experiments, the following antibodies were used: mouse monoclonal antibody targeting IRF3 (Santa Cruz Biotechnology; sc-376455), rabbit monoclonal antibody targeting pIRF3 (Cell Signaling Technology; 4947), mouse monoclonal antibody targeting pSTAT1 (BS Biosciences; 612233), mouse monoclonal antibody targeting beta-actin (Sigma-Aldrich; A5441), and anti-mouse antibodies coupled with horseradish peroxidase (HRP) (GE Healthcare; NA934V).

Human recombinant IFN-β1a (IFN-β1) obtained from Biomol (86421) was used at the indicated concentrations. Recombinant human IFN-λ 2 (IL28A) (300-2K) and IFN-λ 3 (IL-28B) (300-2K) were purchased from Peprotech and were used at the indicated concentrations.

### Western blotting.

Cells were harvested and lysed with 1 radioimmunoprecipitation assay (RIPA) buffer (150 mM sodium chloride, 1.0% Triton X-100, 0.5% sodium deoxycholate, 0.1% sodium dodecyl sulfate [SDS], 50 mM Tris [pH 8.0]) with phosphatase and protease inhibitors (Sigma-Aldrich) for 5 min at 37°C. Lysates were collected and quantified. A total of 10 μg of protein was separated by SDS-PAGE and blotted onto a nitrocellulose membrane by wet blotting (Bio-Rad). Membranes were then blocked with Tris-buffered saline-Tween 20 (TBS-T) containing 5% bovine serum albumin (BSA) for 2 h at room temperature. Primary antibodies were diluted in the same blocking buffer and incubated overnight at 4°C. Membranes were then washed three times with TBS-T for 20 min at room temperature by rocking. Anti-mouse antibodies coupled with horseradish peroxidase were used at a 1:5,000 dilution in blocking buffer, followed by incubation at room temperature for 1 h with rocking. Membranes were washed three times and HRP detection was carried out. HRP detection reagent (GE Healthcare) was mixed 1:1, followed by incubation at room temperature for 5 min. Membranes were exposed to film and developed.

### Indirect immunofluorescence assay.

T84 cells were seeded on iBIDI glass bottom 8-well chamber slides 24 h prior to infection. At the indicated times postinfection, cells were fixed in 2% paraformaldehyde (PFA) for 20 min at room temperature. Cells were washed and permeabilized in 0.5% Triton-X for 15 min at room temperature. BSA (3%)–phosphate-buffered saline (PBS) was used for blocking for 30 min at 37°C. Mouse monoclonal antibody against rotavirus capsid VP6 (Santa Cruz) was used at 1:500 diluted in 1% BSA-PBS and incubated for 1 h at room temperature. Cells were washed in 1× PBS three times and incubated with Alexa Fluor 647 secondary antibodies (Thermo Fisher Scientific) at 1:1,000 and 4′,6-diamidino-2-phenylindole (DAPI) for 30 min at room temperature. Cells were washed in 1× PBS three times and maintained in PBS. Cells were imaged with a Nikon/Andor spinning-disc confocal microscope.

### RNA isolation, cDNA, and qPCR.

RNA was harvested from cells using an RNeasy RNA extraction kit (Qiagen) as per the manufacturer’s instructions. cDNA was made using iSCRIPT reverse transcriptase (Bio-Rad) from 200 ng of total RNA as per the manufacturer’s instructions. qRT-PCR was performed using iTaq SYBR green (Bio-Rad) as per the manufacturer’s instructions. The TBP gene was used as a normalizing gene. Primer used are listed in Table 1.

**TABLE 1:** Sequences of qRT-PCR primers^*a*^

Gene	Forward sequence (5′–3′)	Reverse sequence (5′–3′)
TBP	CCACTCACAGACTCTCACAAC	CTGCGGTACAATCCCAGAACT
IFN-β1	GCCGCATTGACCATCTAT	GTCTCATTCCAGCCAGTG
IFN-λ1	GCAGGTTCAAATCTCTGTCAC	AAGACAGGAGAGCTGCAACTC
IFN-λ2/3	GCCACATAGCCCAGTTCAAG	TGGGAGAGGATATGGTGCAG
Mx1	GAGCTGTTCTCCTGCACCTC	CTCCCACTCCCTGAAATCTG

a All genes are human genes.

### Detection of IFNs in supernatant.

HEK-Blue reporter cell lines (see above) were seeded in FBS-inactivated DMEM/F-12 at a density of 30,000 cells per well in 96-well plates 1 day before the experiment. Supernatants of rotavirus-infected cells were added to HEK-Blue cells for 24 h, and the levels of secreted embryonic alkaline phosphatase (SEAP) were measured using QUANTI-Blue (InvivoGen; rep-qbs). Since IFN-α/β reporter HEK 293 cells are also able to respond to IFN-λ, cells were transfected with a previously generated CRISPR KO vector targeting the IFNLR1 (ref popi), generating reporter cell lines that can sense only IFN-β1.

### Live-cell fluorescence microscopy and image analysis.

T84 cells were seeded in 48-well plates at full confluency 24 h before experiments. Cells were infected as previously described, and the spread of viruses and activation of fluorescent reporters were imaged over time using a Celldiscoverer 7 (Zeiss). Cells were kept at 37°C and 5% CO_2_ during all experiments. For the spread of rotavirus, UnaG was imaged with a 470-nm light-emitting diode (LED) lamp at 30% laser power and 300-ms exposure time. H2B-mCherry was imaged with a 590-nm LED lamp at 50% laser power and 300-ms exposure time. For visualization of activation of the pMx1-mCherry reporter, H2B-mTurquoise2 was imaged using a 420-nm LED lamp at 50% laser power and 300 ms, UnaG using a 511-nm LED lamp at 50% and 300 ms, and pMx1-mCherry with a 590-nm LED lamp at 60% laser power and 500 ms. Quantifications of infection and quantification of Mx1-mCherry-positive cells were carried out using ilastik (https://www.ilastik.org) to generate object masks using the H2B-tagged nuclei as a reference. The basal fluorescence of the reporters was subtracted from infected or treated samples in Fiji (https://fiji.sc) based on the levels found in mock-treated samples. To determine the values used to separate positive from negative cells, the mean intensity of fluorescence found in nuclei was measured in mock-treated and treated samples. CellProfiler (https://cellprofiler.org) was then used to measure the mean intensity of fluorescence in all objects found in the masks (nuclei) under all conditions, and positive cells were quantified using the threshold values obtained from Fiji.

### Statistical analyses.

Statistical analyses were performed using one-way analysis of variance (ANOVA) with Dunnett’s multiple-comparison test using the GraphPad Prism software package.
